# GAS6/TAM Pathway Signaling in Hemostasis and Thrombosis

**DOI:** 10.3389/fmed.2018.00137

**Published:** 2018-05-09

**Authors:** Luke A. Law, Douglas K. Graham, Jorge Di Paola, Brian R. Branchford

**Affiliations:** ^1^Department of Anesthesiology, Mayo Clinic, Rochester, MN, United States; ^2^Section of Hematology/Oncology, Department of Pediatrics, Emory University, Atlanta, GA, United States; ^3^Section of Hematology/Oncology, Department of Pediatrics, University of Colorado School of Medicine, Aurora, CO, United States; ^4^University of Colorado Hemophilia and Thrombosis Center, Aurora, CO, United States

**Keywords:** GAS6, TYRO3, AXL, MERTK, platelet activation, signaling pathways

## Abstract

The GAS6/TYRO3-AXL-MERTK (TAM) signaling pathway is essential for full and sustained platelet activation, as well as thrombus stabilization. Inhibition of this pathway decreases platelet aggregation, shape change, clot retraction, aggregate formation under flow conditions, and surface expression of activation markers. Transgenic mice deficient in GAS6, or any of the TAM family of receptors that engage this ligand, exhibit *in vivo* protection against arterial and venous thrombosis but do not demonstrate either spontaneous or prolonged bleeding compared to their wild-type counterparts. Comparable results are observed in wild-type mice treated with pharmacological inhibitors of the GAS6-TAM pathway. Thus, GAS6/TAM inhibition offers an attractive novel therapeutic option that may allow for a moderate reduction in platelet activation and decreased thrombosis while still permitting the primary hemostatic function of platelet plug formation.

## Introduction

The burden of disease from thrombosis is high, with 1 out of every 6 deaths in the United States caused by coronary heart disease and approximately 690,000 ischemic strokes occurring per year (1). Current anti-platelet therapies are complicated by hemorrhagic side effects and interpatient response variability (2), demonstrating a need to explore new pathways that may yield safer and/or more effective therapies.

One such alternative pathway for the modulation of thrombosis and hemostasis is the TAM family of receptors tyrosine kinases (TYRO3, AXL, and MERTK) and their ligands, including growth arrest-specific gene 6 (GAS6) and Protein S. It has been shown that inhibition of the GAS6/TAM pathway decreases platelet activation responses and protects mice against arterial and venous thrombosis, without increasing bleeding (3, 4) thereby representing a promising therapeutic target for novel anti-platelet agents. This pathway is also active in a variety of benign and malignant cell types with functions including regulation of inflammation (5, 6), phagocytosis of apoptotic cells and cellular debris (5), platelet stabilization (3, 7, 8), maintenance of vascular smooth-muscle homeostasis (6, 9–11), spermatogenesis (12), maintenance of the retina (13), and cancer progression (14).

The abrogation of TAM signaling, as evidenced by studying transgenic mice deficient in one or more receptors, is associated with additional biological effects including significantly increased inflammatory response, impaired clearance of cellular debris and apoptotic cells, and systemic autoimmunity (6, 15, 16). Additionally, deleterious *Mertk* mutations are associated with the development of retinitis pigmentosa in humans, (17–19) a finding also noted in canine (20) and murine models (13). Rodent studies have evaluated the effect of expressing the paralog receptor *Tyro3* (21) or gene therapy with human *MERTK* (22) to abrogate the disease, and recent human studies have involved translational read-through inducing drugs (23). Further information regarding the effects of GAS6/TAM signaling absence or inhibition is shown in Table 1.

**Table 1:** Effects of various Gas6/TAM inhibition strategies.

**Strategy**	**Mechanism of action**	**Mouse phenotype**	**Aggregation**	**Aggregate formation under flow**	**Activation marker expression or ADP release (granule release)**	**Platelet spreading**	**Clot retraction**	**Arterial thrombosis model**	**Venous thrombosis model**	**Hemostasis**
*GAS6* ^−/−^	N/A	Vascular defects, normal hemostasis without spontaneous bleeding (3)	Impaired (3)	Small, unstable (26)	Decreased (3)			Protected (3)	Protected (3)	Normal initial hemostasis, but transient rebleeding (7)
*TYRO3* ^−/−^	N/A	Neurologic Disorders, normal hemostais without spontaneous bleeding (7)	Impaired (7)	Small, unstable (26)	Decreased (7)	Impaired, but not permanently abrogated (7)	Slowed (7)		Protected (7)	Normal initial hemostasis, but transient rebleeding (7)
*AXL*^−/−^	N/A	Vascular Defects, normal hemostais without spontaneous bleeding (7)	Impaired (7)	Small, unstable (26)	Decreased (7)	Impaired, but not permanently abrogated (7)	Slowed (7)		Protected (7)	Normal initial hemostasis, but transient rebleeding (7)
*MERTK*^−/−^	N/A	Autoimmune defects, normal hemostais without spontaneous bleeding (7, 77)	Impaired (7, 77)	Small, unstable (26)	Decreased (7)	Impaired, but not permanently abrogated (7)	Slowed (7)	Protected (77)	Protected (7, 77)	Normal initial hemostasis (77), but transient rebleeding (7)
Triple-null *(Tyro3* ^−/−^*Axl*^−/−^*Mertk*^−/−^)	N/A	Inflammation and all individual null disorders, anti-phospholipid antibodies (16), blindness (16) enlarged spleens (16), small testes, and defective spermatogenesis (16)								Prone to recurrent thrombosis and hemorrhage (61), thrombocytopenia and impaired hemostasis (79)
Anti-GAS6 antibody	Ligand trap	No spontaneous bleeding (3)	Impaired (3)						Protected (3)	
sAxl Axl ECD	Ligand trap	N/A	Impaired (7)						Protected (7)	Normal hemostasis time (7)
sMer	Ligand trap	No spontaneous bleeding (80)	Impaired (80)						Protected (80)	
UNC2025	Receptor-specific inhibitor	N/A	Low (81)	Unstable (81)	Low (81)	Decreased (81)	N/A	Decreased duration of occlusion (81)	Increased survival (81)	Similar to control (81)

This mini-review, however, focuses solely on the role of the GAS6/TAM signaling in hemostasis and thrombosis. Prior to any suggestion of GAS6/TAM-modulation as a reasonable clinical trial candidate for anti-platelet effect, much work remains to be done to establish dose-dependent organ-specific effects to better define any “off-target” considerations.

Platelet exposure to agonists including von Willebrand factor, collagen, thrombin, thromboxane A2, and adenosine diphosphate (ADP) leads to platelet activation and recruitment of subsequent platelets in a dynamic process known as “inside-out” signaling (24, 25). This process involves the rapid engagement of cell surface receptors that activate signal transduction pathways involving Src family kinases, Akt, phosphoinositide 3- kinases (PI3Ks), and the ITAM signaling pathway, ultimately leading to intracellular calcium mobilization and conversion of the fibrinogen receptor, integrin α_IIb_β_3_ from an inactive (low-affinity) to an active (high-affinity) conformation. Upon fibrinogen engagement to the activated α_IIb_β_3_ “outside-in” (24) signaling occurs promoting platelet spreading, clot retraction, granule secretion, and stable adhesion. The combination of these processes leads to a stable clot formation. It has previously been shown that “outside-in” signaling can be inhibited by interruption of the GAS6/TAM receptor pathway (7).

## GAS6 structure and receptor interactions

### GAS6 (ligand)

**Figure 1:**
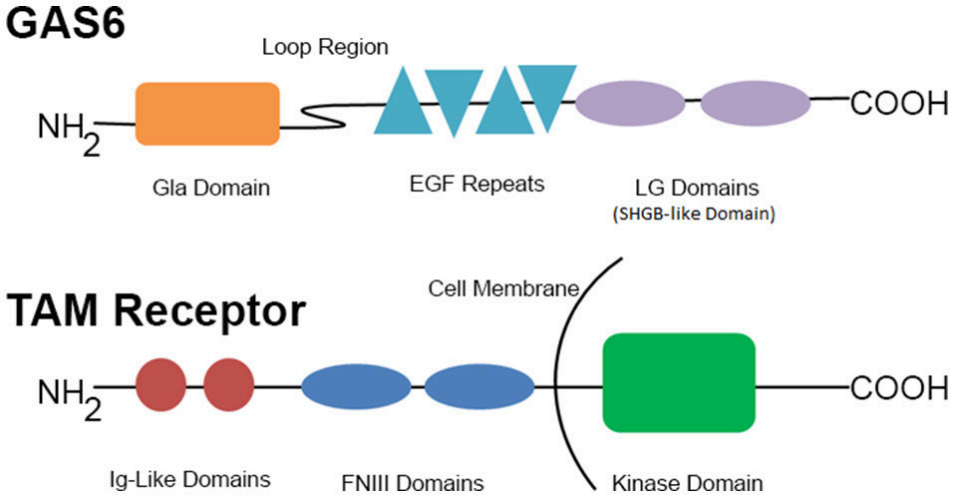
GAS6 and representative TAM family receptor. This schematic represents the components of GAS6 and the TAM receptors. EGF, epidermal growth factor; FNIII, fibronectin III; Ig, immunoglobulin; LG, laminin G-like.

GAS6 (Figure 1), a 75 kDa product of the growth arrest-specific gene 6 (*Gas6*), is a vitamin K-dependent growth factor expressed in many cell types including vascular smooth muscle cells, mesangial cells, endothelial cells, bone marrow stromal cells, and macrophages (26–28), as well as in human platelets and plasma (3, 26, 29). Cellular secretion of GAS6 in the vasculature is primarily autocrine and is stimulated by several platelet agonists such as thrombin or collagen (3), as well as erythropoietin (30), and the pro-inflammatory cytokine TGFβ (31).

Other components of this signaling pathway are also active and may contribute to the pathologic state known as thromboinflammation (32, 33). GAS6 levels are elevated in inflammatory conditions such as sepsis/systemic inflammatory response syndrome (34), systemic lupus erythematosus (35–37), and atherosclerotic plaques in vascular smooth muscle cells (31). GAS6 universally exhibits anti-inflammatory effects through downregulation of TNFα, interleukin-6, interferon γ, and intercellular adhesion molecule-1 expression, playing a significant role in preserving immune homeostasis (15, 27, 31, 38, 39). In addition to blocking platelet activation and protecting mice from thrombosis (3), inhibition or deficiency of *Gas6* has also been shown to prevent liver inflammation, steatohepatitis, and hepatic fibrosis (40) but enhanced colitis-related tumorigenesis (41) in murine models.

**Figure 2:**
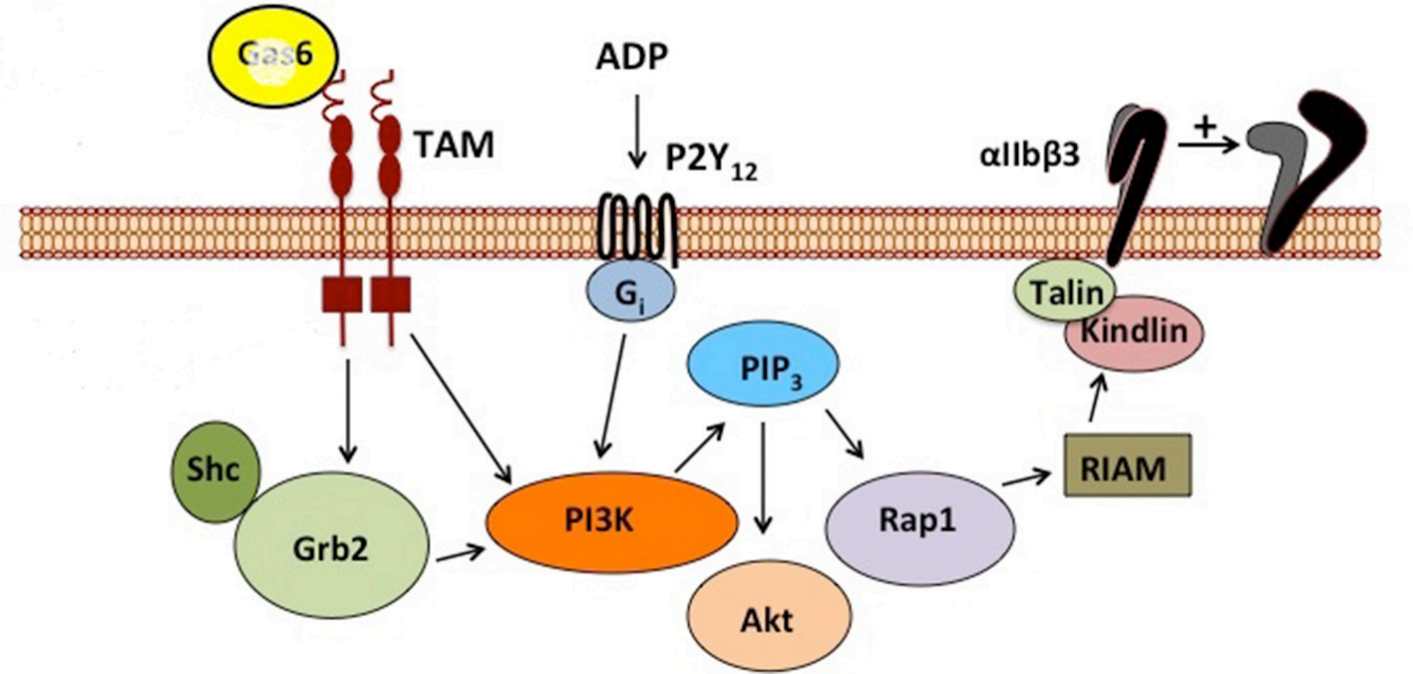
Schematic representation of GAS6/TAM signaling pathway. This figure depicts the signal transduction cascade initiated by GAS6 binding to TYRO3, AXL, or MERTK and the complementary contribution of the ADP/P2Y signaling pathway.

Once secreted, GAS6 primarily binds to the TAM family receptor tyrosine on the platelet surface (42) by the C-terminal sex hormone binding globulin (SHBG)-like domain composed of two laminin G domains (Figure 1). This binding triggers dimerization and autophosphorylation (43–45), of these receptors and subsequent activation of the downstream signaling molecules PI3K (46, 47), Rap1 (47–49), and Akt (50–54). As seen in Figure 2, the activation of PI3K/Akt leads to phosphorylation of the cytoplasmic tail of the β_3_ integrin, promoting propagation and amplification of “outside-in” signaling (7, 55, 56), resulting in shape change, clot retraction, and subsequent platelet plug stabilization.

The N-terminal Gla domain of GAS6 (Figure 1) can also undergo calcium-dependent structural transformations allowing for high-affinity binding to phosphatidylserine (PtdS) residues (54, 57–60) exposed on the surface of nearby cells in response to cell activation, stress, and apoptosis (27). This allows GAS6 to target a wide variety of injured or activated cells in clinical settings such as endothelial cell remodeling (11), regulation of innate immunity (12, 61), vascular smooth-muscle homeostasis (9, 62), erythropoiesis (30), and survival regulation of tumor cells from mesenchymal, epithelial and hematopoietic origins (63, 64). Additionally, GAS6 bridges membrane-bound PtdS and TAM receptors (27, 54, 58, 59). The Gla domain of GAS6 [also involved in the regulation of osteoclast function (65, 66) and oligodendrocyte survival (67–69)] is connected to a disulfide-bridged loop, which, in turn, connects to four epidermal growth factor domains and a SHBG-like domain (Figure 1). Protein S, a negative regulator of the clotting cascade, is a close structural analog of GAS6, but has a disulfide-bridged loop that interacts with activated protein C following serine protease cleavage—to which GAS6 is insensitive due to structural constraints.

GAS6 does not appear to be a primary effector of platelet activation (70), but enhances and extends the platelet activation response triggered by ADP and other agonists through modulation of “outside-in” signaling via the α_IIb_β_3_ integrin (3, 26) and regulation of granule secretion. It has been proposed that autocrine signaling in platelets is possible through release of GAS6 from α-granules (3, 7, 71–73). The precise source of GAS6 in human blood is not well established. Most studies indicate the presence of GAS6 in human plasma with levels varying from 15 to 65 μg/L (26, 74–76). This variation in levels of GAS6 in plasma had no correlation with extent of platelet activation in humans (70). While at least one study did not reveal physiologically relevant amounts of GAS6 in human platelets (75), others have demonstrated the presence of GAS6 mRNA (3, 77) as well as the protein itself at low concentrations (20 μg/L, equivalent to 5ng per 10^9^ platelets) by various techniques, including immunoelectron microscopy and Western blots (26). While GAS6 levels in murine platelets are 6-fold higher than in human platelets, the plasma levels are comparable (78).

### Other ligands

In addition to GAS6, other ligands are known to stimulate the TAM receptors, including Protein S (82), Tubby, Tubby-like protein (TULP1), and Galectin-3. While GAS6 and Protein S are both vitamin K dependent proteins and share approximately 43% amino acid sequence identity and have the same domain structure (83). Protein S has been shown to be capable of binding TYRO3 (65) and MERTK (84), but has not yet been found to exhibit affinity for AXL (16). Tubby and TULP1 facilitate retinal pigment epithelium and macrophage phagocytosis through MERTK binding (85), while Galectin-3 is a MERTK-specific “eat-me” signal that stimulates phagocytosis of apoptotic cells and cellular debris by macrophages and retinal pigment epithelial cells (86) but is also upregulated after tissue injury such as myocardial infarction or in both acute and chronic liver injury, as described in a recent review (87).

### Gas6 receptors (TAM subfamily of tyrosine kinases)

GAS6 binds to, and promotes tyrosine phosphorylation of, the single transmembrane tyrosine kinases of the TAM receptor subfamily (7, 71). Members of this receptor family exhibit a common structure determined by two extracellular N-terminal immunoglobulin-like domains and two fibronectin-III-like domains followed by a tyrosine kinase domain residing at the C-terminal (cytoplasmic) portion, as seen in Figure 1 (88, 89).

Although, of the TAM receptors, AXL has the highest *in vitro* demonstrated affinity for GAS6 with Kd = 1.0 nMol/L (followed by TYRO3 with roughly equal affinity) (90) it has been shown in some studies that MERTK (Kd at least 10-fold lower) (91, 92), is the only GAS6 receptor detected on platelets (whether human or murine) (77). Other studies, however, have not only reported the presence of AXL in platelets using immunoelectron microscopy, but also noted that labeling patterns suggest changes in location after stimulation of the platelets (26). A comprehensive review of this signaling pathway describes platelet expression of all three TAM receptors (56). Taken together, these varied results demonstrate a distinct lack of current consensus regarding expression in platelets and further research is warranted.

## Genetic deficiency of GAS6 or TAM receptor(s)

*Gas*6^−/−^ mice exhibit normal development, produce adequate-sized litters, and appear phenotypically normal (3). They have coagulation factor levels and clotting times within the normal range and exhibit normal platelet and megakaryocyte counts as well as normal platelet ultra-structural morphology (3). *Gas6*^−/−^ mice do not demonstrate spontaneous bleeding or thrombosis. In fact, these mice are protected from thrombosis, and their platelets create smaller aggregates under flow conditions in comparison to their wild type (WT) counterparts (an effect reversible with the addition of recombinant human GAS6) (26).

Additionally, *Gas6*^−/−^ mice exhibit decreased aggregation in response to low agonist concentrations [ADP 2.5–10 μM, collagen 2 μg/mL, and U46619 (thromboxane A_2_ analog) 10 μM] (3). At the same low agonist concentration, platelets from *Gas6*^−/−^ mice showed evidence of normal shape change but did not aggregate to the same degree as platelets from WT mice. At high agonist concentrations (ADP 50 μM, collagen 5–15 μg/mL, and U46619 100 μM), irreversible aggregation was comparable between *Gas6*^−/−^ and WT mice. Interestingly, with stimulation by a strong agonist such as thrombin, aggregation was comparable between *Gas6*^−/−^ and WT platelets, but electron microscopy showed that *Gas6*^−/−^ platelets were less densely packed, incompletely degranulated and made fewer connections with neighboring platelets (3). Testing the hypothesis that GAS6 is involved in cancer-induced coagulopathy, Aghourian et al. induced inferior vena cava thrombi with FeCl_3_ in both WT and *GAS6*^−/−^ mice with lung carcinoma (93). The *Gas6*^−/−^ mice formed much smaller thrombi than their WT counterparts, however larger thrombi were seen in *Gas6*^−/−^ mice by injecting recombinant GAS6, indicating GAS6 inhibition may have anti-thrombotic effects in the setting of lung cancer (93).

*Gas6*^−/−^ mice also exhibit decreased thrombosis *in* vivo. In a stasis-induced venous thrombosis model *Gas6*^−/−^ mice developed significantly smaller thrombi than did WT mice (3). In a Rose Bengal model of arterial thrombosis, the *Gas6*^−/−^ mice formed thrombi 60% smaller than did their WT counterparts (3). In an induced pulmonary embolism (PE) model histological examination failed to demonstrated the extensive PEs in the *Gas6*^−/−^ mice that were consistently present in WT mice (3).

Genetic deficiencies of individual receptors for GAS6 also result in decreased platelet activation responses and protection from thrombosis *in vivo*. Loss of any one of the TAM receptors was sufficient to protect mice from collagen/epinephrine- and stasis-induced thromboembolism (7) without evidence of major hemorrhagic side effects, though *GAS6*^−/−^ mice did have a tendency to re-bleed after tail clipping (7, 8) likely due to inability to form stable aggregates. After an initial platelet activation response to thrombin, mice deficient in one of the TAM receptors show significant reduction in clot retraction. For example, *Mertk*^−/−^ mice demonstrate significantly lower platelet aggregation in response to low-moderate concentrations of agonist as compared to wild type mice (7). Additionally, no second wave of platelet aggregation is observed, likely due to impaired granule release. *Mertk*^−/−^ mice were also protected from carotid artery occlusion in a ferric chloride induced injury model showing increased time to complete occlusion of the vessel as well as increased artery patency after 30 min. Further, bleeding time via tail clipping does not differ significantly between *Mertk* ^−/−^ and WT mice.

Interestingly, a more significant difference between WT and *Mertk*^−/−^ was observed at lower agonist concentrations (2.5 μg/mL collagen, 10 μM U46619, and 0.25 mMoL/L PAR4 thrombin receptor agonist peptide AYPGKF) (7) indicating that strong stimulation from higher agonist concentrations may overcome the functional deficit, similar to that described *Gas6*^−/−^ mice. Finally, *Mertk*^−/−^ mice are viable, fertile and do not exhibit significant differences in litter size, mouse weight, cell counts in any lineage or coagulation tests such as the prothrombin time and partial thromboplastin time when compared to WT mice. In addition, the binding of *Gas6*^−/−^ platelets to fibrinogen on initial activation with various agonists was not impaired, hence “inside-out” platelet signaling and subsequent activation of α_IIb_β_3_, does not appear to be affected. However, α_IIb_β_3_ tyrosine phosphorylation as well as Akt phosphorylation are significantly decreased and platelet spreading was impaired in mice deficient in one of the TAM receptors. Taken together, these data indicate that absence of any of the TAM receptors in platelets appears to cause inhibition of the “outside-in” signaling pathway. The GAS6/TAM pathway is not a primary activator of platelets, as this signal transduction cascade does not stimulate platelets by itself, but rather augments and potentiates activation processes initiated by other agonists (26, 81). Its inhibition (with or without additional antagonism of the ADP/P2Y12 pathway), therefore, has a mild anti-platelet and anti-thrombotic effect, with the benefit of minimal observable hemorrhagic effect.

*Tyro3*^−/−^ mice likewise demonstrate reduced thrombus formation and decreased platelet aggregation stability (but not initial aggregation), due to decreased outside-in signaling and subsequent limitation of platelet granule secretion (7). Effects on inflammation in this strain are not well described, but neurologic effects of *Tyro3* knockout include neural degeneration with seizures and paralysis (12).

*Axl*^−/−^ mice exhibit hematologic effects such as reduced thrombus formation and decreased platelet aggregate stability, similar to the *Tyro3*^−/−^ and *Mertk*^−/−^ mice (7). Effects on inflammation include an increase in apoptosis and enhanced inflammation in the CNS because of delayed removal of myelin debris during experimental autoimmune encephalomyelitis (94). Additionally, these mice exhibit elevated vascular permeability and impaired vascular remodeling (including Increase in CD45+ cells and decrease in VSMC, macrophages, and neutrophils) (44).

Indeed, genetic disruption of any one of the three receptors is associated with a significant reduction in expression of the other two, and a total reduction in the platelet's ability to bind to and activate from GAS6 stimulation (7). Finally, decreased alpha granule secretion has been reported in *Tyro3*^−/−^, *Axl*^−/−^, and *Mertk*^−/−^ mice (8).

Transgenic murine models with genetic deletion of all three TAM receptors have been described with platelet function inhibition similar to those mice with single receptor deletions. Additionally, they are prone to recurrent thrombosis and hemorrhage (61), thrombocytopenia and impaired hemostasis (79). They also exhibit elevated inflammation/auto-immunity, anti-phospholipid antibodies, blindness, enlarged spleens, small testes, and defective spermatogenesis (16).

## Pharmacologic inhibition of the GAS6/TAM pathway

### Anti-GAS6 antibodies and GAS6 traps

Anti-GAS6 antibodies have been shown to be effective inhibitors of platelet activation. Mice pre-treated with anti-GAS6 antibodies displayed protection from PE in a similar manner as that seen with *Gas6*^−/−^ mice, and neither group showed evidence of increased bleeding (3). Additionally, it was demonstrated that GAS6 neutralizing antibodies inhibited aggregation in human washed platelets (3, 71). Not surprisingly, inhibition of aggregation with anti-GAS6 antibodies was observed at low agonist concentrations which was reversed with increasing concentration of agonists (71).

Trapping of GAS6 with a recombinant extracellular domain of AXL (hAxl-EC-Fc) resulted in a dose-dependent blockade of platelet aggregation in response to ADP, but had no effect in the absence of ADP (7). These mice were protected from collagen/epinephrine induced PE in a manner similar to the *GAS6*^−/−^ mice, and showed no spontaneous bleeding or increase in bleeding times (7). Purified preparations of cell-surface cleavage products comprising plasma-derived soluble extracellular domains of MERTK (sMer) (80) and AXL(sAxl) (29) also have been shown to bind and sequester GAS6, resulting in decreased platelet aggregation and protection of mice from collagen-epinephrine induced PE.

### Receptor-specific small molecule inhibitors

Recent studies have shown that MERTK-specific small molecule inhibitors UNC2025 and UNC2881 inactivate the MERTK tyrosine kinase domain, disrupting subsequent signaling (4, 81, 95). Of note, UNC2025 is equipotent against MERTK and FLT3, with 50-fold greater selectivity in cell-based assays than for AXL (95). Since FLT3 expression has not been reported in human or murine megakaryocytes or platelets, the antiplatelet effect of this compound is unlikely to be mediated through FLT3 inhibition.

Mice treated with these molecules demonstrated protection from PE in the collagen/epinephrine model, and the average patency time of the carotid after injury with FeCl_3_ was significantly increased (81). Additionally, treated mice did not show increased bleeding by tail clipping or spontaneous bleeding. Platelets from mice treated with these inhibitors also demonstrated decreased activation responses, decreased surface activation markers, and reduced stability of aggregates formed under physiologic flow conditions. UNC2025 caused a significant decrease in p-selectin positive platelets and total platelet counts (CD41 positive) at vascular injury locations via a laser intravital vascular microscopy model (81). These findings suggest that MERTK-specific small molecule inhibitors cause decreased accumulation without affecting platelet adhesion at sites of vascular injury.

The inhibition of platelet activation and protection from thrombosis afforded by the GAS6/TAM antagonists described above is encouraging but should be considered with caution. The limited predictive value of murine tail bleed hemostasis models are well-known (96) and may underestimate the bleeding risk imparted by certain compounds, such as the anti-factor VIII therapeutic antibody LE2E9 that prevented thrombosis without increased bleeding risk in animal models but increased hemorrhagic side effects compared to controls in a human therapeutic trial (97).

## GAS6/TAM antagonists combined with ADP/P2Y inhibitors

Washed human platelets pre-treated with a combination of UNC2025 and ADP/P2Y inhibitors (ADPis) demonstrated a statistically significant synergistic decrease in platelet aggregation and mice treated with the same combination exhibited a longer time until artery occlusion and shorter duration of occlusion in a FeCl_3_-induced carotid artery injury model than would be expected if combining the effects of the two agents independently, (81) suggesting a synergistic interaction. Similarly, the mice given a combination of low dose ADPi and UNC2025 were better protected against mortality in the collagen/epinephrine model than mice given either agent alone. As opposed to increased bleeding noted after tail clip in the high dose ADPi group, mice treated with a combination of ADPis and UNC2025 demonstrated no increased spontaneous bleeding or tail clip-induced bleeding.

The synergistic effect noted in these studies likely relates to the combination of effects on different areas of a thrombus that may disrupt its stable formation. When considered in the context of a hierarchical description of thrombus development proposed by Stalker et al. GAS6/TAM inhibitors appear to affect the thrombin-stimulated platelet core while ADP/P2Y inhibitors act on the shell (98).

## Conclusion

Inhibition of the Gas6/TAM pathway leads to decreased platelet activation responses *in vitro* and protects mice from arterial or venous thrombosis *in vivo*. As mice with knockout of either the ligand or any of the TAM receptors are known to have normal hemostasis labs and do not exhibit spontaneous bleeding or prolonged time to cessation in bleeding models, this pathway represents an attractive potential target for novel anti-platelet agents with reduced risk of hemorrhagic complications compared to current agents. Further research is needed to address the potential effects of deletion or inhibition of this pathway outside of the hemostatic system.

## Author contributions

All authors listed have made a substantial, direct and intellectual contribution to the work, and approved it for publication.

### Conflict of interest statement

The authors declare that the research was conducted in the absence of any commercial or financial relationships that could be construed as a potential conflict of interest.
